# Comparative Transcriptome Analysis Reveals New lncRNAs Responding to Salt Stress in Sweet Sorghum

**DOI:** 10.3389/fbioe.2020.00331

**Published:** 2020-04-15

**Authors:** Xi Sun, Hongxiang Zheng, Jinlu Li, Luning Liu, Xiansheng Zhang, Na Sui

**Affiliations:** ^1^Shandong Provincial Key Laboratory of Plant Stress, College of Life Sciences, Shandong Normal University, Jinan, China; ^2^State Key Laboratory of Crop Biology, College of Life Sciences, Shandong Agricultural University, Tai’an, China; ^3^Institute of Integrative Biology, University of Liverpool, Liverpool, United Kingdom; ^4^College of Marine Life Sciences, Ocean University of China, Qingdao, China

**Keywords:** sweet sorghum, non-coding regulatory, lncRNA, ceRNA network, salt tolerance

## Abstract

Long non-coding RNAs (lncRNAs) can enhance plant stress resistance by regulating the expression of functional genes. Sweet sorghum is a salt-tolerant energy crop. However, little is known about how lncRNAs in sweet sorghum respond to salt stress. In this study, we identified 126 and 133 differentially expressed lncRNAs in the salt-tolerant M-81E and the salt-sensitive Roma strains, respectively. Salt stress induced three new lncRNAs in M-81E and inhibited two new lncRNAs in Roma. These lncRNAs included lncRNA13472, lncRNA11310, lncRNA2846, lncRNA26929, and lncRNA14798, which potentially function as competitive endogenous RNAs (ceRNAs) that influence plant responses to salt stress by regulating the expression of target genes related to ion transport, protein modification, transcriptional regulation, and material synthesis and transport. Additionally, M-81E had a more complex ceRNA network than Roma. This study provides new information regarding lncRNAs and the complex regulatory network underlying salt-stress responses in sweet sorghum.

## Introduction

Salt stress is now one of the main abiotic factors constraining agricultural productivity worldwide. More than 6% of the arable soil worldwide is affected by salinity (Song and Wang, 2015; Yuan et al., 2016). When the local topsoil contains an excessive amount of soluble salts, it forms a saline-alkali soil, which damages plants to varying degrees, possibly leading to death. Salt stress may adversely affect cells by leading to ionic, osmotic, oxidative, and nutrient stresses, and can substantially decrease crop quality and quantity by disrupting plant growth and metabolism (Feng et al., 2014; Sui and Han, 2014; Yang et al., 2017; Cui et al., 2018).

Numerous recent studies have indicated that non-coding RNA can enhance the stress resistance of plants by regulating the expression of some functional genes (Xin et al., 2011; Mondal et al., 2018). Non-coding RNAs can be divided into small non-coding RNA (sncRNA) and long non-coding RNA (lncRNA) based on their length. MicroRNA (miRNA), which is an important type of sncRNA, is a single-stranded small RNA with only 21 nucleotides (nt) in plants (Seitz, 2009). Mature miRNAs form miRNA-mediated silencing complexes (MIRISCs) with AGOs and silence gene expression by cleaving and degrading the target genes (Eric, 2011). In contrast, lncRNAs are non-coding RNAs that are longer than 200 nt, and there is increasing evidence that lncRNAs are potential regulatory molecules (Mercer et al., 2009). Specifically, lncRNAs can influence gene expression at the transcriptional, post-transcriptional, epigenetic, and other levels to modulate various biological processes that enable, plants to resist abiotic and biotic stresses. There is currently considerable interest in lncRNAs among molecular biologists and geneticists. A previous study proved, that lncRNAs can function as competitive endogenous RNAs (ceRNAs) to competitively bind to the same miRNAs as mRNAs through miRNA response elements (MREs), thereby modifying target gene expression and mediating the resistance to various stresses (Yvonne et al., 2014). In plants, the standard for target mimics is much stricter than that in animals, and the positive position of the sequence must not contain paired bases (resulting in protrusions). Recent studies have revealed that lncRNAs can alter the expression of target genes through *cis*- or *trans*-regulation and can be cleaved by miRNAs to generate siRNAs to silence target genes (Wang et al., 2018; Fu et al., 2019; Rizvi and Dhusia, 2019). Additionally, some natural antisense lncRNAs help regulate target gene expression (Swiezewski et al., 2009; Huanca-Mamani et al., 2018). Although ceRNAs have been well studied in animals (Qu et al., 2015; Meng et al., 2018), relatively little is known about ceRNAs in plants, beyond the current research on the associated regulatory mechanism.

Sweet sorghum [*Sorghum bicolor* (L.) Moench], which is also known as sugar sorghum, is an annual C4 crop (family Gramineae) with a high biomass and tolerance to adverse conditions, including salinity, drought, and flooding (Guo et al., 2018). In an earlier study, we determined that the differences between the salt-tolerant sweet sorghum line (M-81E) and the salt-sensitive line (Roma) are partly due to the diversity in the regulation of certain genes in specific pathways (Yang et al., 2018). However, it remains unclear whether non-coding RNAs affect the salt tolerance of sweet sorghum. In this study, we analyzed the root transcriptomes of sweet sorghum lines M-81E and Roma via high-throughput Illumina RNA sequencing (RNA-seq). We compared the transcriptomes of these lines under salt stress conditions, by examining the differential expression of lncRNAs, miRNAs, and mRNAs, and constructed the ceRNA networks related to differentially expressed mRNAs. The results presented herein may be useful for elucidating the complex regulatory network involving non-coding RNAs underlying the tolerance of sweet sorghum to salt stress.

## Materials and Methods

### Plant Materials and Salt Treatments

On the basis of one of our previous studies (Sui et al., 2015), we selected the salt-tolerant sweet sorghum line M-81E and salt-sensitive line Roma as the experimental materials for the RNA-seq analysis. The two sweet sorghum lines were cultured at 28 ± 3°C (day/night) with 15-h photoperiod (light intensity of 600 μmol m^–2^ s^–1^) and 70% relative humidity as described by Yang et al. (2018). An earlier investigation proved that 150 mM NaCl is an appropriate concentration for detecting significant differences in physiological parameters between M-81E and Roma (Sui et al., 2015). Therefore, starting at the three-leaf stage, the plants were treated with nutrient solutions containing 0 or 150 mM NaCl. In the experimental group, the NaCl concentration was increased by 50 mM every 12 h up to the final concentration. The plants were analyzed in the subsequent experiments after a 24-h exposure to 150 mM NaCl for.

### Whole Transcriptome Library Construction and High-Throughput Sequencing

Total RNA was extracted with the TRIzol reagent (Invitrogen, CA, United States) according to the manufacturer’s protocol. The total RNA quantity and purity were analyzed with a 2100 Bioanalyzer and the RNA 6000 Nano LabChip Kit (Agilent, CA, United S), with an RIN number >7.0. Libraries of small RNAs (<50 nt) were prepared from approximately 1 μg total RNA with the TruSeq Small RNA Sample Preparation Kit (Illumina, San Diego, CA, United States). Approximately 10 μg total RNA was treated with the Epicentre Ribo-Zero Gold Kit (Illumina, San Diego, CA, United States) to eliminate ribosomal RNA, after which the mRNA was purified and fragmented, with the mRNA-Seq sample preparation kit (Illumina, San Diego, CA, United States). Reverse transcribed cleavage of RNA fragments was used to generate de-RNA strand-specific de-RNA >200 nt. Single-end and paired-end sequencing analyses were performed with the Illumina HiSeq 2500 and Illumina HiSeq 4000 systems, respectively, at LC-BIO (Hangzhou, China). The SE50 strategy was used for the small RNA library and the 150PE on-board strategy was used for the de-RNA chain-specific library.

### Assembly and Identification of lncRNAs

The raw sequencing reads were cleaned with Cutadapt, whereas FastQC^1^ was used to verify the sequence quality. The reads were mapped to the assembled transcripts of the sweet sorghum genomic sequence with Bowtie2 and TopHat2, and then assembled with StringTie (Pertea et al., 2015). These reads were screened to filter out the known mRNAs encoding proteins, transcripts <200 bp, and transcripts with fewer than <3 reads (Langmead and Salzberg, 2012; Kim et al., 2013). The coding potential calculator (CPC), coding-non-coding index (CNCI) and Pfam were then used to predict the transcripts with coding potential, and all transcripts with a CPC score <−1 and a CNCI score <0 were discarded. Finally, the remaining transcripts with one or more exons and with class codes (i, j, o, u, and x) were defined as lncRNAs (Kong et al., 2007; Finn et al., 2009). The class codes represented the following: where i, a transfrag falling entirely within a reference intron; j, potentially novel isoform (fragment) with at least one splice junction shared with a reference transcript; o, generic exonic overlap with a reference transcript; u, unknown and intergenic transcript; and x, exonic overlap with a reference sequence on the opposite strand (Figure 2B). The raw sequence data has been uploaded to NCBI under accession number GSE145748^2^.

### Analysis of Differentially Expressed Genes and Differentially Expressed lncRNAs

StringTie was used to calculate the fragments per kilobase of exon model per million mapped fragments (FPKM) values, which were used to represent the gene and lncRNA expression levels (Pertea et al., 2015). The differentially expressed genes (DEGs) and differentially expressed lncRNAs (DELs) were identified with the R package Ballgown with log_2_(fold-change) > 1 or <−1 and *p* < 0.05 (Frazee et al., 2015).

### Predicting and Analyzing lncRNA-miRNA-mRNA Relationship Pairs

According to our RNA-seq data, the principle of scoring prediction based on the complementary pairing principle of sequences, using TargetFinder software to predict miRNA targets (Allen et al., 2005; Fahlgren and Carrington, 2010). The predictions were based on the following rules: (1) the middle of the miRNA sequence is complementary to the base of the lncRNA, but this position produces a 3–5-nt bulge on the lncRNA; (2) no more than four total mismatches are allowed in the non-intermediate region of each miRNA, and there should be no more than two consecutive nt mismatches; and (3) no protrusions are allowed in the non-intermediate region of the miRNA. Cytoscape^3^ was then used to construct a putative network of interactions.

### Quantitative Real-Time PCR Analysis

The relative expression levels of lncRNAs, miRNAs, and mRNAs were analyzed by quantitative real-time PCR (qRT-PCR). A total plant RNA extraction kit (Hua Yueyang, Beijing, China) was used to prepare RNA samples for the M-81E and Roma plants treated with 0 and 150 mM NaCl for 24 h (Sui et al., 2015). Total RNA was isolated from the primary root. The RNA was quantified with the Nanodrop-ND-1000 Spectrophotometer (Thermo Fisher Scientific, Wilmington, DE, United States). The sweet sorghum actin gene was used as an endogenous reference gene for lncRNAs and mRNAs, and *U6* was used as an endogenous reference gene for miRNAs. The stem-loop method was used to reverse transcribe the miRNAs. Primer sequences are listed in Supplementary Table 1.

## Results

### High-Throughput Sequencing and DEG Analysis

The whole transcriptome sequencing analysis was completed with three biological replicates for each sample (M-81E-CK1, M-81E-CK2, M-81E-CK3, M-81E-salt1, M-81E-salt2, M-81E-salt3, Roma-CK1, Roma-CK2, Roma-CK3, Roma-salt1, Roma-salt2, and Roma-salt3). Single-end sequencing with the Illumina HiSeq 2500 system and paired-end sequencing with the Illumina HiSeq 4000 system were used to generate more than 363,000,000 raw reads from the control and salt-stressed M-81E and Roma samples. Approximately 54.5 and 53.7 G of raw and valid reads, respectively, were detected (i.e., more than 98% of the raw reads were valid reads; Supplementary Table 2). To assess the quality of the RNA-seq data, Fast QC was used to assign a quality score (Q) to each base in the reads with a phred-like algorithm (Ewing et al., 1998). The analysis confirmed that the data were highly reliable.

**FIGURE 1:**
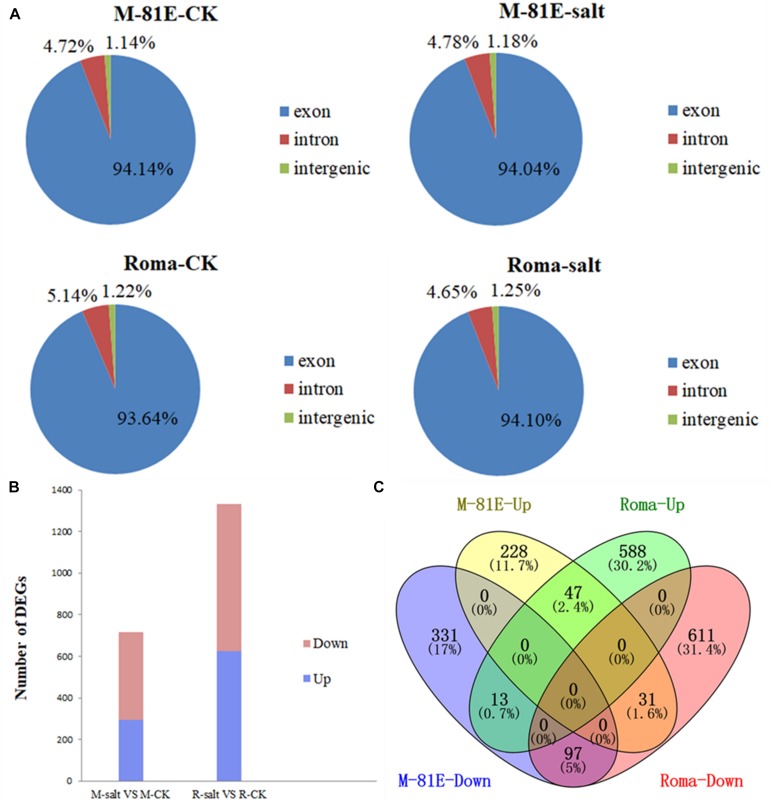
Unique reads mapped to various genomic regions, and identification and characterization of DEGs in M-81E and Roma between salt stress and control samples. **(A)** Unique reads mapped to various genomic regions. **(B)** Numbers of DEGs. **(C)** Venn diagram showing DEGs typically expressed in M-81E and Roma samples.

After mapping to the sweet sorghum genome, approximately 86.14, 84.93, 80.62, and 87.53% of the reads were mapped in the M-81E-CK, M-81E-salt, Roma-CK, and Roma-salt samples, respectively, with more than 74.59, 72.44, 69.04, and 76.17% uniquely mapped reads, respectively (Supplementary Table 2). The set of unique mapped reads was mapped to exons (94.14% of M-81E-CK, 94.04% of M-81E-salt, 93.64% of Roma-CK, and 94.10% of Roma-salt), introns (4.72% of M-81E-CK, 4.78% of M-81E-salt, 5.14% of Roma-CK, and 4.65% of Roma-salt), and intergenic regions (1.14% of M-81E-CK, 1.18% of M-81E-salt, 1.22% of Roma-CK, and 1.25% of Roma-salt) (Figure 1A). The results revealed that lncRNAs in sweet sorghum are predominantly derived from exon transcripts.

**FIGURE 2:**
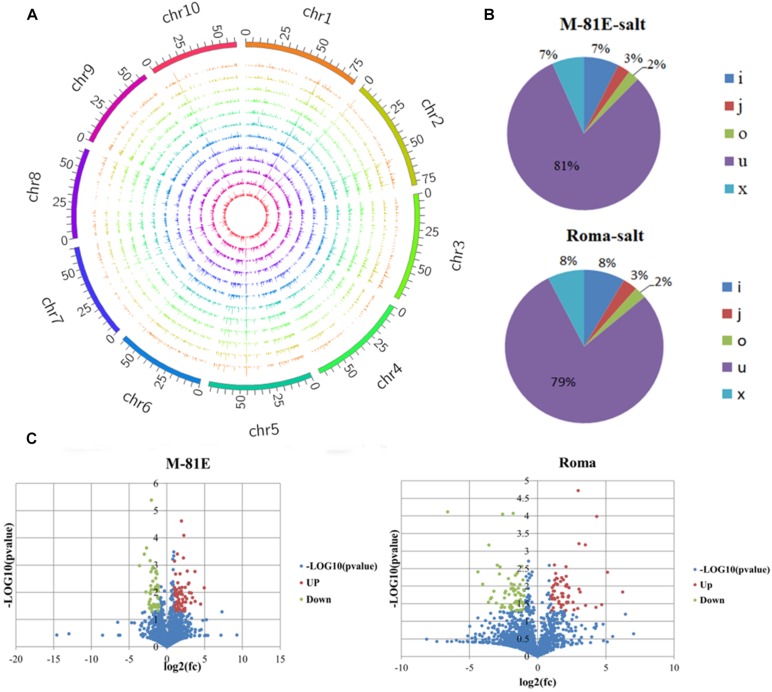
Identification and characterization of DELs. **(A)** Distribution of lncRNA on the chromosomes. **(B)** Unique reads mapped to various genomic regions. I, a transfrag falling entirely within a reference intron; j, potentially novel isoform (fragment) with at least one splice junction shared with a reference transcript; o, generic exonic overlap with a reference transcript; u, unknown, intergenic transcript; x, exonic overlap with reference on the opposite strand. **(C)** Number of DELs identified in M-81E-CK vs M-81E-salt and Roma-CK vs Roma-salt. Among them, the blue points indicate the DELs (–1 > log_2_(fc) > 1), the green points indicate the downward adjustment of DELs (log_2_(fc) < –1, *P* ≤ 0.05), and the red points indicate the upward adjustment of DELs(log_2_(fc) > 1, *P* ≤ 0.05).

After calculating the expression level of each gene (FPKM), 714 genes were identified as differentially expressed between M-81E-CK and M-81E-salt [log_2_(fold-change) ≤−1 or ≥1, *p* < 0.05]. Among these DEGs, 293 and 421 were up-regulated and down-regulated, respectively. Additionally, 1,331 genes were differentially expressed between Roma-CK and Roma-salt, of which 623 were up-regulated and 708 were down-regulated (Figure 1B).

A Venn diagram of the DEGs indicated that 228 genes in M-81E and 588 genes in Roma were uniquely up-regulated, whereas 47 up-regulated genes were co-expressed in M-81E and Roma. Among the down-regulated genes, 331 were M-81E-specific, 611 were Roma-specific, and 97 were co-expressed in both M-81E and Roma. Moreover, 31 genes were down-regulated in Roma but up-regulated in M-81E, whereas 13 genes were down-regulated in M-81E but up-regulated in Roma (Figure 1C).

The DEGs between the control and NaCl-treated plants were functionally annotated based on a gene ontology (GO) analysis (Supplementary Figure S1). In M-81E, 21,754 genes were assigned 900 Level-2 GO terms, of which 11 DEGs with a specific GO term were related to salt-stress responses. In Roma, 21,754 genes were assigned to 1,324 Level-2 GO terms, of which 31 DEGs with a specific GO term were associated with salt-stress responses. In the biological process category, the “biological process” and “regulation of transcription, DNA-templated” terms were the most prominent in M-81E and Roma. The most highly enriched terms in the cellular component category for M-81E and Roma were “nuclear” and “plasma membrane.” In the molecular function category, “molecular function” and “ATP binding” were the most represented GO terms in M-81E, but “molecular function” and “protein binding” were the most represented GO terms in Roma (Supplementary Figure S1). These results implied that salt stress may affect plants by changing the plasma membrane structure and function.

### Identification and Characterization of lncRNAs

After an analysis of all transcripts from the RNA-seq data as well as the functional annotation and filtration, 2,176 unique lncRNAs were identified for the M-81E and Roma samples. These lncRNAs were evenly distributed on the 10 sweet sorghum chromosomes (Figure 2A). Moreover, we compared the distribution of the transcript length and number, exon number and expression levels of lncRNAs and mRNAs (Supplementary Figure S2). The mRNA transcripts were not only longer than the lncRNA transcripts, and they were but also more abundant and expressed at higher level. The lncRNAs were mostly enriched in segments with transcripts shorter than 300 nt and mostly comprised one exon. These results differ from those of a previous study (Yang et al., 2019), likely because of the apparent species specificity of these sweet sorghum lncRNAs. Additionally, the lncRNA open reading frames were less abundant and shorter than the mRNA open reading frames (Supplementary Figure S3).

On the basis of the genomic locations of lncRNAs in M-81E, 7% of the lncRNAs had an exonic overlap with a reference sequence on the opposite strand (x); 7% of the lncRNAs were transfrags entirely within a reference intron (i); 3% of the lncRNAs were potentially novel isoforms (fragments) with at least one splice junction shared with a reference transcript (j); 2% of the lncRNAs had a generic exonic overlap with a reference transcript (o); and 81% of the lncRNAs were unknown and intergenic transcripts (u). In Roma, 8% of the lncRNAs had an exonic overlap with a reference sequence on the opposite strand (x); 8% of the lncRNAs were transfrags entirely within a reference intron (i); 3% of the lncRNAs were potentially novel isoforms (fragments) with at least one splice junction shared with a reference transcript (j); 2% of the lncRNAs had a generic exonic overlap with a reference transcript (o); and 79% of the lncRNAs were unknown and intergenic transcripts (u) (Figure 2B).

Of these 2,176 lncRNAs, 126 DELs [log_2_(fold-change) ≤−1 or ≥1, *p* < 0.05] were identified by comparing the M-81E-salt and M-81E-CK samples. These DELs included 71 up-regulated and 55 down-regulated lncRNAs (Figure 2C). A total of 133 DELs [log_2_(fold-change) ≤−1 or ≥1, *p* < 0.05] were identified in the Roma samples, including 68 up-regulated and 65 down-regulated lncRNAs (Figure 2C).

#### Analysis of lncRNA-Related ceRNA Networks in Sweet Sorghum Roots and a Functional Enrichment Analysis of Related DEGs

Previous studies proved that lncRNAs can function as ceRNAs and regulate gene expression at the post-transcriptional level (Zhang et al., 2018; He et al., 2019). Additionally, lncRNAs serve as target mimics that interact with miRNAs thereby preventing miRNAs from completely degrading their targets (Wu et al., 2013; Deng et al., 2018; Meng et al., 2018). To assess the regulatory patterns of non-coding RNA-related ceRNA interactions in sweet sorghum roots, lncRNA-miRNA-mRNA pairs were predicted in the treated and control of M-81E and Roma samples. Cytoscape^4^ was then used to construct a putative network of interactions. Consequently, 672 lncRNA-miRNA-mRNA interactions were predicted in the global ceRNA network, of which 477 and 195 were predicted in M-81E and Roma, respectively (Supplementary Figure S4). Furthermore, 13 lncRNAs were involved in the 672 lncRNA-miRNA-mRNA regulatory networks, of which seven were in M-81E and six were in Roma.

**FIGURE 3:**
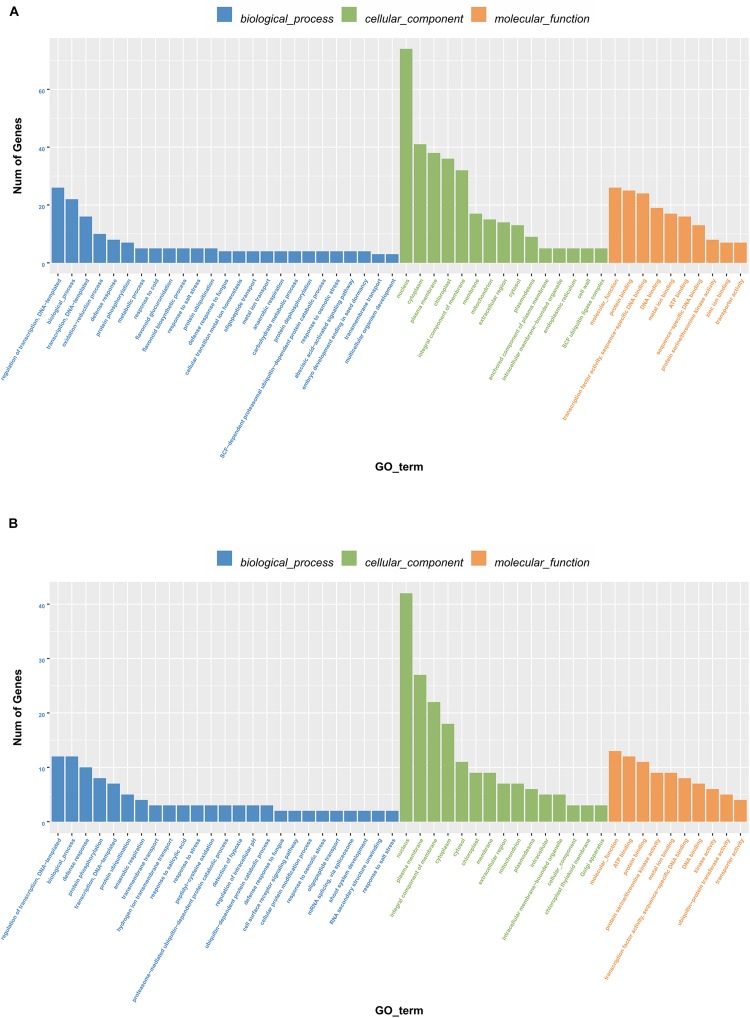
Gene ontology enrichment of 13 lncRNAs as ceRNA regulated protein-coding genes in M-81E **(A)** and Roma **(B)**. The y-axis represents the number of genes and the x-axis represents the GO functional group.

To further elucidate how the DELs confer salt tolerance to sweet sorghum, we functionally characterized the genes regulated by the 13 lncRNAs based on a GO analysis (Figure 3). In the biological process category, addition to “transcriptional regulation,” the protein-encoding genes in M-81E were enriched with other terms, including “redox process” and “defense reaction,” whereas in Roma, the terms “defense reaction” and “protein phosphorylation” were enriched. In the cellular component category, besides the term “nucleus” and “cytoplasm” were the most enriched terms in M-81E, whereas, “plasma membrane” was the most enriched term in Roma. In the molecular function category, “protein binding” and “ATP binding” were the most enriched GO terms in M-81E and Roma.

**FIGURE 4:**
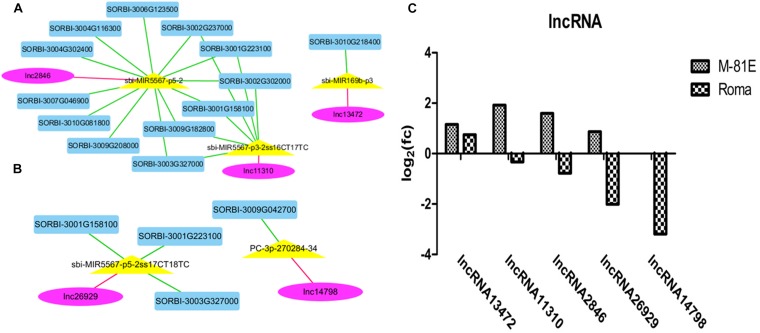
A ceRNA network enriched in salt response pathways in M-81E **(A)** and Roma **(B)**. Pink, yellow, and blue nodes represent lncRNAs, miRNAs, and mRNAs, respectively. Green edges represent miRNA-target interactions, while pink edges represent a competitive relationship. **(C)** Expression level of five unknown lncRNAs in M-81E and Roma.

After a colocalization and expression analysis with Cytoscape and the construction of a network of interactions, five DELs (three in M-81E and two in Roma) were detected from among the 13 lncRNAs possibly related to salt responses. The five DELs (lncRNA13472, lncRNA11310, lncRNA2846, lncRNA26929, and lncRNA14798) were identified as modulators of miRNAs that regulate five miRNAs and 14 target genes (Figures 4A,B). LncRNA13472 might compete with *SORBI_3010G218400* for the binding to sbi-MIR169b-p3. Moreover, lncRNA11310 might compete with *SORBI_3001G158100*, *SORBI_3001G223100*, *SORBI_ 3002G237000*, *SORBI_3002G302000*, *SORBI_3003G327000*, and *SORBI_3009G182800* for the binding to sbi-MIR5567-p3-2ss16CT17TC. Additionally, lncRNA2846 might compete with *SORBI_3001G158100*, *SORBI_3001G223100*, *SORBI_3002G237 000*, *SORBI_3002G302000*, *SORBI_3003G327000*, *SORBI_3004 G116300*, *SORBI_3004G302400*, *SORBI_3006G123500*, *SORBI_ 3007G046900*, *SORBI_3009G182800*, *SORBI_3009G208000*, and *SORBI_3010G081800* for the binding to sbi-MIR5567-p5-2. In contrast, lncRNA26929 might compete with *SORBI_3003 G327000*, *SORBI_3001G158100*, and *SORBI_3001G223100* for the binding to sbi-MIR5567-p5-2ss17CT18TC, whereas, lncRNA14798 might compete with *SORBI_3009G042700* for the binding to PC-3p-270284-34.

The expression levels of the five DELs differed between M-81E and Roma. Although lncRNA13472 expression was up-regulated in both M-81E and Roma, its expression level was higher in M-81E than in Roma. The expression of lncRNA11310, lncRNA2846, and lncRNA26929 increased in M-81E and decreased in Roma. The lncRNA14798 expression level was unchanged in M-81E, but decreased in Roma (Figure 4C).

**FIGURE 5:**
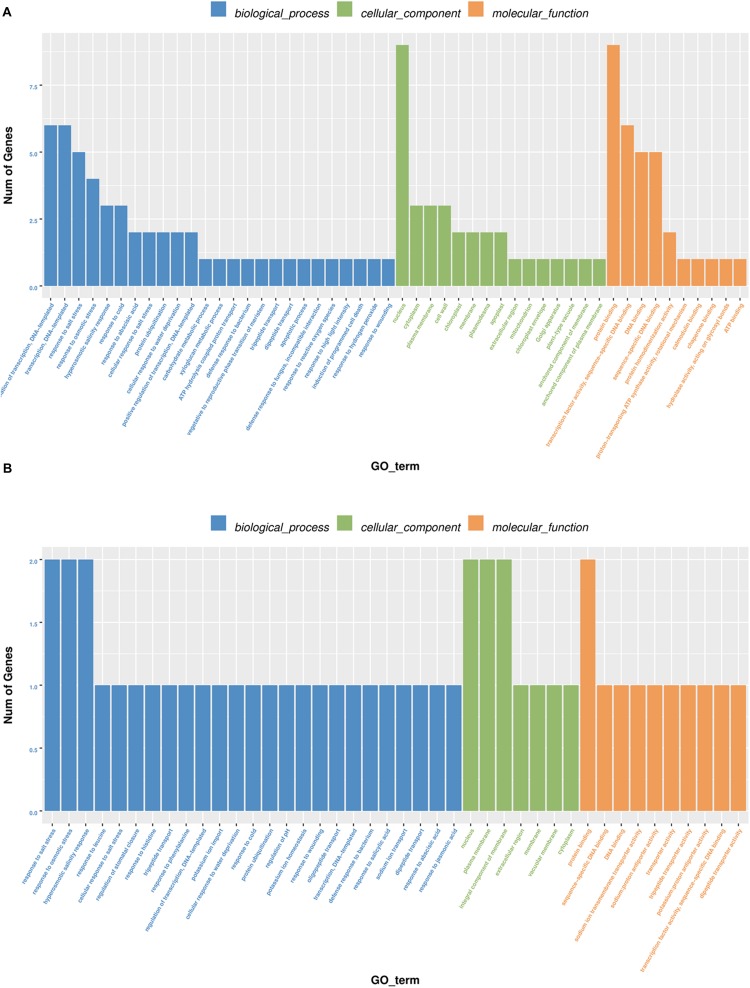
Gene ontology enrichment of five lncRNAs as ceRNA regulated protein-coding genes in M-81E **(A)** and in Roma **(B)**. The results summarize the three main categories: biological processes, molecular functions, and cellular components. The y-axis represents the number of genes and the x-axis represents the GO functional group.

To demonstrate how the ceRNA network helps regulate the salt tolerance of sweet sorghum roots, we performed a functional enrichment analysis of the 14 target proteins based on GO annotations. The results revealed that the protein-encoding genes were mainly enriched with stress-related terms, including “regulation of transcription,” “response to salt stress,” “nucleus,” “protein binding,” and “transport activity” (Figure 5). These findings suggested that in response to salt stress, lncRNAs may function as ceRNAs and compete with miRNAs as binding partners for mRNA, which may regulate the metabolism of several protein-encoding genes involved in important biological processes.

### qRT-PCR Validation of Differentially Expressed Transcripts From RNA-seq

**FIGURE 6:**
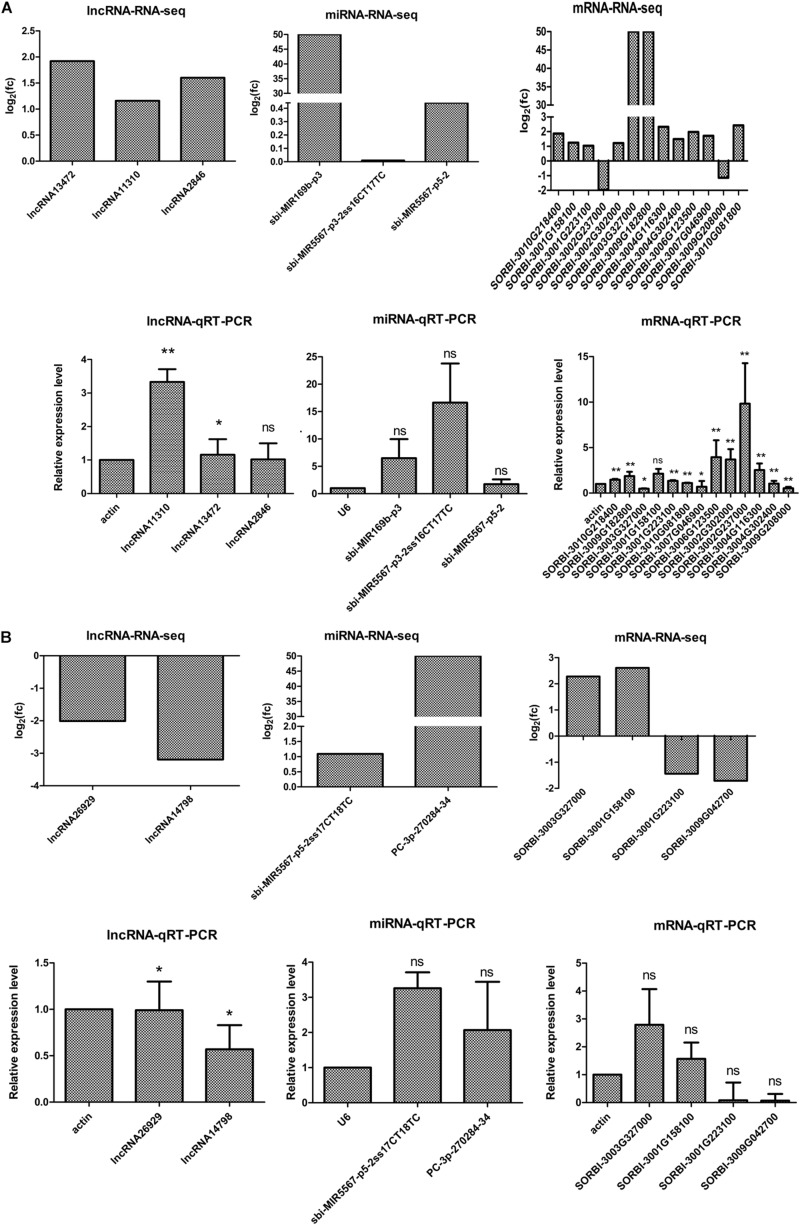
Quantitative RT-PCR analysis and RNA-seq results of ceRNA regulatory networks in M-81E **(A)** and Roma **(B)**. Three biological replicates were prepared for each of these samples. Actin is the endogenous reference gene for lncRNAs and mRNAs, and *U6* is the endogenous reference gene for miRNAs. The y-axis shows the relative expression levels analyzed by qRT-PCR. Based on three biological replicates, each repeated three times, the standard deviation of the relative expression levels (*n* = 3) is indicated by error bars. Significant differences between each RNA and internal reference were also assessed separately (*p* < 0.05). ** indicates *p* < 0.01, * indicates *p* < 0.05, and ns indicates *p* > 0.05.

To verify the putative relationships among the 5 DELs, 5 miRNAs, and 14 DEGs, their expression levels were examined by qRT-PCR. The qRT-PCR data revealed that in M-81E, the expression levels of 3 DELs (lncRNA13472, lncRNA11310, and lncRNA2846), 3 miRNAs (sbi-MIR169b-p3, sbi-MIR5567-p3-2ss16CT17TC, and sbi-MIR5567-p5-2), and 10 DEGs (*SORBI_3010G218400, SORBI_3001G158100, SORBI_ 3001G223100, SORBI_3002G302000, SORBI_3003G327000, SORBI_3009G182800, SORBI_3004G116300, SORBI_3004G302 400, SORBI_3006G123500, SORBI_3007G046900, and SORBI_ 3010G081800*) were up-regulated. In contrast the expression levels of two DEGs (*SORBI_3002G237000* and *SORBI_3009 G208000*) were down-regulated. Similarly, in Roma, the expression levels of two miRNAs (sbi-MIR5567-p5-2ss17 CT18TC and PC-3p-270284-34) and two DEGs (*SORBI_ 3003G327000* and *SORBI_3001G158100*) were up-regulated, whereas the expression levels of two DELs (lncNRA26929 and lncNRA14798), and two DEGs (*SORBI_3001G223100* and *SORBI_3001G042700*) were down-regulated. The qRT-PCR results were consistent with the RNA-seq data, which confirmed the reliability of the RNA-seq analysis (Figure 6).

## Discussion

Salt stress induces ion toxicity, osmotic stress and oxidative stress, which can adversely affect plant metabolism and growth (Kumar et al., 2018; Liang et al., 2018). Plants respond and adapt to highly saline environments at the transcriptional and post-transcriptional levels (Tariq and Paszkowski, 2004; Feller et al., 2011). The regulation at the transcriptional level is mainly mediated by transcription factors, DNA methylation, histone modifications and other processes that activate or inhibit the expression of specific gene (Zhang et al., 2016). At the post-transcriptional level, the regulation is mainly achieved via the alternative splicing of RNA, as well as RNA methylation and multiple RNA–RNA interactions (Wassenegger et al., 1994; Zhu et al., 2007; Bardou et al., 2014). We previously confirmed that a high-salt treatment inhibits Roma roots growth starting at 24 h, but inhibits M-81E roots growth starting at 36 h. Under saline conditions, many functional genes are differentially expressed between M-81E and Roma (Yang et al., 2018). These functional genes are important for the salt tolerance of sweet sorghum, but it is unknown whether other regulatory pathways are also involved?

Recent studies have indicated lncRNAs can regulate physiological metabolism as well as growth and development by contributing to the regulation of histone modifications, nucleic acid structural modifications, nucleic acid methylations and RNA–RNA interactions (Matsui and Seki, 2019; Qin and Xiong, 2019). In cotton, lncRNAs are active in salt-stress responses (Deng et al., 2018). However, this study focused on *cis*-acting regulation in which lncRNAs function as ubiquitous regulators. Wang et al. (2015) described the differences in the number of lncRNAs in the roots and leaves of *Medicago truncatula* in response to salt stress. Specifically, they detected considerably more lncRNAs in roots than in leaves, suggesting that lncRNAs are especially active in plant roots. Furthermore, lncRNAs are relatively enriched in ceRNAs and may be one of the major sources of ceRNAs (Li et al., 2019). There is limited available information regarding the regulatory functions of lncRNAs in salt-stressed sweet sorghum. In the present study, we performed an RNA-seq analysis of M-81E and Roma roots treated with NaCl to explore how lncRNAs function as ceRNAs during sweet sorghum responses to salt stress.

After annotating, characterizing, and analyzing the RNA-seq data, we predicted the ceRNA relationships of the DELs, and identified 477 and 195 lncRNA-miRNA-mRNA relationships in M-81E and Roma, respectively (Supplementary Figure S2). These results imply that the competitive interaction of lncRNAs in the ceRNA network may be a new regulatory factor in response to salt stress. We detected substantially more interactions in M-81E than in Roma, suggesting that M-81E has more precise and complex ceRNA regulatory mechanisms that mediate responses salinity.

The expression levels of five lncRNAs varied between M-81E and Roma (Figure 4), further suggesting that there are more complex networks in M-81E. These lncRNAs were predicted to compete with 14 DEGs for the binding to five miRNAs (Figures 4A,B). Notably, sbi-MIR5567 was detected as a sorghum-specific miRNA (Zhang et al., 2011). The homologs of sbi-MIR5567 (sbi-MIR5567-p3-2ss16CT17TC, sbi-MIR5567-p5-2, and sbi-MIR5567-p5-2ss17CT18TC) detected in this study also have important functions in both M-81E and Roma. How they participate in the complex ceRNA regulatory networks under salt stress condition remains unknown and will need to be determined in further studies.

**FIGURE 7:**
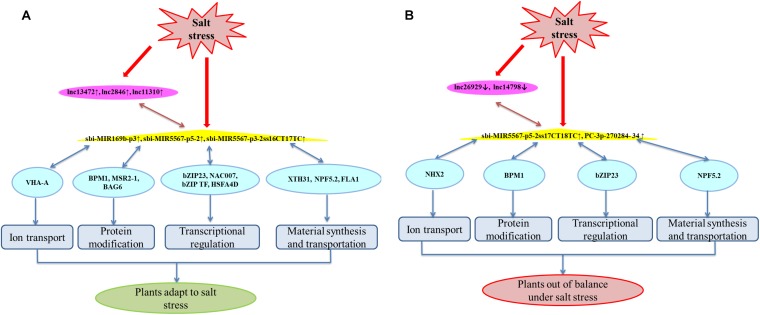
A hypothetical model for ceRNA regulatory networks in M-81E **(A)** and Roma **(B)**. Pink, yellow, and blue nodes represent lncRNAs, miRNAs, and mRNAs, respectively. Blue edges represent miRNA-target interactions, pink edges represent a competitive relationship.

The lncRNA-miRNA-mRNA relationships and the roles of the 14 DEGs were analyzed in greater detail. The miRNAs target many transcription factors, signaling factors, and transporter-encoding genes involved in salt-stress responses (Zhang et al., 2013). Compared with that of Roma, the ceRNA regulatory network in M-81E involves more pathways. These DELs participate in sweet sorghum responses to high salinity primarily by regulating ion transport, protein modifications, transcription, and material synthesis and transport (Figure 7). The DEGs mainly encode proton pumps, transport proteins, specific enzymes, and transcription factors (Supplementary Tables 3–5). Among these DEGs, *SORBI_3010G218400* encodes the catalytic subunit A of the V-type proton ATPase (VHA-A). Earlier investigations revealed that V-ATPase is an ATP-dependent proton pump that is involved in the transmembrane transport of protons and contributes to salt-stress responses (Powell et al., 2000; Vera-Estrella et al., 2005). In the current study, the *SORBI_3010G218400* expression level increased in M-81E, which likely helps establish a transmembrane proton gradient. The expression of sbi-MIR169b can be down-regulated under drought conditions (Maor, 2017). However, we observed that its expression level was up-regulated (Figure 6A). On the basis of our data, we predict that *SORBI_3010G218400* competes with lncRNA13472 for the binding to sbi-MIR169b-p3. The up-regulated expression of *SORBI_3010G218400* and sbi-MIR169b-p3 will promote the binding of lncRNA13472 to sbi-MIR169b, which is consistent with our results (Figure 6A).

Analyses of protein modifications, transcription, and material synthesis and transport in M-81E produced similar results. Both *SORBI_3001G158100* and *SORBI_3007G046900* encode BTB/POZ and MATH domain-containing protein 1 (BPM1). In the cytoplasm, this protein may serve as a substrate-specific adapter, bind to the E3 ubiquitin-protein ligase complex, and mediate the ubiquitination of target proteins for the subsequent degradation by the 26S proteasome (Chen et al., 2013). Members of the BPM family regulate ABA responses and can control the opening and closing of stomata, making them crucial for plant development and stress responses (Chen et al., 2013). Additionally, *SORBI_3006G123500* is an important antioxidant gene (Moskovitz et al., 1997; Oh et al., 2005) encoding methionine sulfoxide reductase A2-1 (MSRA2-1), which participates in oxidative-stress responses and is induced by salt stress in plants. Moreover, *SORBI_3004G116300* encodes Bcl-2*-*associated athanogene (BAG6), which selectively promotes the proteasomal degradation of mislocalized proteins via ubiquitination (Leznicki and High, 2012). Kang et al. (2006) confirmed that *AtBAG6* is a stress-regulated calmodulin-binding protein associated with programmed cell death in plants. The *SORBI_3003G327000* and *SORBI_3009G182800* genes encode basic leucine zipper 23 (bZIP23). Previous studies on *Arabidopsis thaliana* indicated that bZIP proteins are involved in plant responses to drought and high salinity as well as the ABA-dependent signal transduction pathway (Uno et al., 2000; Kang et al., 2002). The *SORBI_3004G302400* and *SORBI_3010G081800* genes encode NAC domain-containing protein 7 (NAC007) and bZIP transcription factor 23 (bZIP TF 23), respectively. These transcription factors bind to the DNA-binding domain to induce or inhibit target gene expression, thereby improving the salt tolerance and drought resistance of plants (Hu et al., 2006; Hsieh et al., 2010). The *SORBI_3002G302000* gene encodes xyloglucan endotransglucosylase/hydrolase protein 31 (XTH31), which is produced mainly in the roots. This enzyme catalyzes xyloglucan endohydrolysis and/or endotransglycosylation, reassembles xyloglucan polymers, and regulates root hair development in *A. thaliana* (Vissenberg et al., 2001; Cho and Cosgrove, 2002), whereas it responds to drought stress in maize (Wu et al., 2001). The *SORBI_3001G223100* gene encodes NRT1/PTR FAMILY 5.2 (NPF5.2), which is a transporter of diverse substances, including nitrates, chlorides, and phytohormones (Xin et al., 2011; Corratgé-Faillie and Lacombe, 2017). Furthermore, BPM1, bZIP23, XTH31, and NPF5.2 can bind to two miRNAs competing with two lncRNAs. The target genes have a non-negligible role in the sweet sorghum response to salt stress. Accordingly, the regulated regulatory lncRNAs and miRNAs also have important function under saline condition.

In this study, the miRNA binding by the above-mentioned genes and the competitor lncRNAs increased, but the lncRNAs and mRNAs were more highly expressed than the miRNAs in M-81E (Supplementary Tables 3, 4). Therefore, we speculate that the mRNA expression levels in M-81E may be induced by high-salt environments to prevent damages due to salt stress. However, the increase in mRNA expression is accompanied by an increase in the abundance of miRNAs that will bind to and degrade target genes to maintain the original homeostasis. In M-81E, the competitive binding of miRNAs increases because of the up-regulated expression of lncRNAs to maintain the differential expression of specific genes responsive to salt stress. This indirectly enhances the salt tolerance of M-81E. However, the specific underlying mode of action remains to be characterized.

The *SORBI_3009G208000* and *SORBI_3002G237000* expression levels were down-regulated under salt stress conditions, whereas the expression of the corresponding lncRNAs and miRNAs was up-regulated. We speculate that the degradation of miRNAs for target genes may be exacerbated by salt-induced damages, and plants respond to salt stress by up-regulating lncRNA expression to decrease mRNA degradation. The *SORBI_3009G208000* gene encodes the important heat shock transcription factor (HSF) A4d. A recent study demonstrated that HSF interactions with HSPs help mediate plant stress resistance (Fragkostefanakis et al., 2019). In addition to heat stress, plant HSFs are also responsive to drought, oxidative, and biotic stresses (Scharf et al., 2012). Moreover, *SORBI_3002G237000* encodes fasciclin-like arabinogalactan protein 1 (FLA1), which is involved in salt-stress responses (Shi et al., 2003). The expression levels of most wheat genes encoding FLAs are down-regulated following an exposure to abiotic stresses (Faik et al., 2006), which is consistent with our results. The exact roles for these two genes in salt-stressed sweet sorghum remain to be investigated.

Unlike in M-81E, the predicted lncRNA expression levels decreased in Roma, whereas the expression of miRNA was up-regulated. These findings suggest saline conditions increase the degradation of mRNAs in Roma. Furthermore, *SORBI_3009G042700* encodes a sodium/hydrogen exchanger 2-like protein (NHX2), which is a Na^+^, and K^+^/H^+^ antiporter mainly localized to the tonoplast membrane. As an ion transporter related to homeostasis, NHX2 regulates the intracellular ion homeostasis and decreases ionic toxicity in plants due to salt stress (Ueda et al., 2018). An earlier study proved that NHX2 confers stress tolerance by participating in the regulation of K^+^ homeostasis, intracellular pH, and stomatal opening and closing (Barragán et al., 2012). Under salt stress conditions, lncRNA14798 expression was down-regulated in Roma, but the PC-3p-270284-34 expression level was up-regulated. The PC-3p-270284-34 will bind to and degrade *SORBI_3009G042700*, thereby decreasing the abundance of the encoded protein. This may help to explain why Roma is more sensitive to salt stress than M-81E.

## Conclusion

In conclusion, our data revealed five unknown lncRNAs in M-81E and Roma under salt stress conditions. These DELs function as ceRNAs that affect the salt tolerance of sweet sorghum by regulating the transcription of genes encoding proton pumps, transporters, important enzymes, and transcription factors. The salt-tolerant M-81E has more precise and complex ceRNA regulatory mechanisms that control salt-stress responses than the salt-sensitive Roma. Our results may be important for clarifying the salt-tolerance mechanism regulated by non-coding RNAs in crops.

## Data Availability Statement

The datasets used and/or analyzed in the current study are available from the corresponding author on reasonable request.

## Author Contributions

XS and HZ performed the experiments. XS and NS collected the data and carried out all the analyses. XS, HZ, and JL initiated preparation of the manuscript. NS, XZ, and LL conceptualized the idea and revised the manuscript. All authors have read and approved the final manuscript.

## Conflict of Interest

The authors declare that the research was conducted in the absence of any commercial or financial relationships that could be construed as a potential conflict of interest.

## Footnotes

1 http://www.bioinformatics.babraham.ac.uk/projects/fastqc/

2 https://www.ncbi.nlm.nih.gov/geo/query/acc.cgi?acc=GSE145748

3 http://www.cytoscape.org/

4 http://www.cytoscape.org/

## Supplementary Material

FIGURE S1 Functional enrichment analysis of differentially expressed genes in M-81E **(A)** and Roma **(B)**.

FIGURE S2 Comparison of lncRNA and mRNA of the transcript length, number, exon number and expression levels. **(A)** The length of lncRNA and mRNA. The x-axis represents the length of the transcript, and the y-axis represents the percentage of lncRNA and mRNA, respectively. **(B)** The number of lncRNA and mRNA. **(C)** The exon number of lncRNA and mRNA. **(D)** The expression levels of lncRNA and mRNA.

FIGURE S3 Comparison of lncRNA **(A)** and mRNA **(B)** of open reading frames.

FIGURE S4 The global ceRNA network in the two sweet sorghum strains. Red, yellow, and blue nodes represent lncRNAs, miRNAs, and mRNAs, respectively.

TABLE S1 Primer sequences for qRT-PCR of 5 lncRNAs, 5 miRNAs and 14 mRNAs.

TABLE S2 Statistical data of the RNA-seq reads for two samples.

TABLE S3 lncRNA-miRNA-mRNA relationship pairs and functional annotation of DEGs.

TABLE S4 Predicted lncRNAs in ceRNA networks.

TABLE S5 Predicted miRNAs in ceRNA networks.

## Abbreviations

BAG6: Bcl-2-associated athanogene
BPM1: BTB/POZ and MATH domain-containing protein 1
bZIP TF 23: bZIP transcription factor 23
bZIP23: basic leucine zipper 23
ceRNAs: competitive endogenous RNAs
CNCI: coding-non-coding index
CPC: coding potential calculator
DEGs: differentially expressed genes
DELs: differentially expressed lncRNAs
FLA1: fasciclin-like arabinogalactan protein 1
FPKM: fragments per kilobase of exon model per million mapped fragments
GO: gene ontology
(HSF) A4D: heat shock transcription factor A4d
i: a transfrag falling entirely within a reference intron
j: potentially novel isoform (fragment): at least one splice junction is shared with a reference transcript
lncRNA: long non-coding RNAs
MIRISCs: miRNA-mediated silencing complexes
miRNA: MicroRNA
MREs: miRNA response elements
MSRA2-1: methionine sulfoxide reductase A2-1
NAC007: NAC domain-containing protein 7
NHX2: sodium/hydrogen exchanger 2-like
NPF5.2: NRT1/PTR FAMILY 5.2
o: generic exonic overlap with a reference transcript RNA-seq: high-throughput Illumina RNA sequencing
sncRNA: small non-coding RNAs
u, unknown: intergenic transcript
VHA-A: V-type proton ATPase catalytic subunit A
x: exonic overlap with reference on the opposite strand
XTH31: xyloglucan endotransglucosylase/hydrolase protein 31.

## References

[B1] Allen E. Xie Z. Gustafson A. M. Carrington J. C. (2005). microRNA-directed phasing during trans-acting siRNA biogenesis in plants. *Cell* 121 207–221. 10.1016/j.cell.2005.04.004 15851028

[B2] Bardou F. Ariel F. Simpson C. G. Romero-Barrios N. Laporte P. Balzergue S. (2014). Long noncoding RNA modulates alternative splicing regulators in *Arabidopsis*. *Dev. Cell* 30 166–176. 10.1016/j.devcel.2014.06.017 25073154

[B3] Barragán V. Leidi E. O. Andrés Z. Rubio L. De Luca A. Fernández J. A. (2012). Ion exchangers NHX1 and NHX2 mediate active potassium uptake into vacuoles to regulate cell turgor and stomatal function in *Arabidopsis*. *Plant Cell* 24 1127–1142. 10.1105/tpc.111.095273 22438021 PMC3336136

[B4] Chen L. Lee J. H. Weber H. Tohge T. Witt S. Roje S. (2013). *Arabidopsis* BPM proteins function as substrate adaptors to a cullin3-based E3 ligase to affect fatty acid metabolism in plants. *Plant Cell* 25 2253–2264. 10.1105/tpc.112.107292 23792371 PMC3723624

[B5] Cho H.-T. Cosgrove D. J. (2002). Regulation of root hair initiation and expansin gene expression in *Arabidopsis*. *Plant Cell* 14 3237–3253. 10.1105/tpc.006437 12468740 PMC151215

[B6] Corratgé-Faillie C. Lacombe B. (2017). Substrate (un) specificity of *Arabidopsis* NRT1/PTR FAMILY (NPF) proteins. *J. Exp. Bot.* 68 3107–3113. 10.1093/jxb/erw499 28186545

[B7] Cui F. Sui N. Duan G. Liu Y. Han Y. Liu S. (2018). Identification of metabolites and transcripts involved in salt stress and recovery in peanut. *Front. Plant Sci.* 9:217. 10.3389/fpls.2018.00217 29520289 PMC5827294

[B8] Deng F. Zhang X. Wang W. Yuan R. Shen F. (2018). Identification of *Gossypium hirsutum* long non-coding RNAs (lncRNAs) under salt stress. *BMC Plant Biol.* 18:23. 10.1186/s12870-018-1238-0 29370759 PMC5785843

[B9] Eric H. (2011). Gene silencing by microRNAs: contributions of translational repression and mRNA decay. *Nat. Rev. Genet.* 12 99–110. 10.1038/nrg2936 21245828

[B10] Ewing B. Hillier L. Wendl M. C. Green P. (1998). Base-calling of automated sequencer traces usingPhred. I. Accuracy assessment. *Genome Res.* 8 175–185. 10.1101/gr.8.3.175 9521921

[B11] Fahlgren N. Carrington J. C. (2010). “miRNA target prediction in plants,” in *Plant MicroRNAs*, eds Meyers B. Green P. (Totowa: Humana Press), 51–57. 10.1007/978-1-60327-005-2_4 19802588

[B12] Faik A. Abouzouhair J. Sarhan F. (2006). Putative fasciclin-like arabinogalactan-proteins (FLA) in wheat (*Triticum aestivum*) and rice (*Oryza sativa*): identification and bioinformatic analyses. *Mol. Genet. Genomics* 276 478–494. 10.1007/s00438-006-0159-z 16944204

[B13] Feller A. Machemer K. Braun E. L. Grotewold E. (2011). Evolutionary and comparative analysis of MYB and bHLH plant transcription factors. *Plant J.* 66 94–116. 10.1111/j.1365-313X.2010.04459.x 21443626

[B14] Feng Z. Deng Y. Fan H. Sun Q. Sui N. Wang B. (2014). Effects of NaCl stress on the growth and photosynthetic characteristics of *Ulmus pumila* L. seedlings in sand culture. *Photosynthetica* 52 313–320. 10.1007/s11099-014-0032-y

[B15] Finn R. Mistry J. Tate J. Coggill P. Heger A. Pollington J. (2009). The Pfam protein families database. *Nucleic Acids Res.* 32 D138–D141. 10.1093/nar/gkh121 PMC308855 14681378

[B16] Fragkostefanakis S. Simm S. El−Shershaby A. Hu Y. Bublak D. Mesihovic A. (2019). The repressor and co−activator HsfB1 regulates the major heat stress transcription factors in tomato. *Plant Cell Environ.* 42 874–890. 10.1111/pce.13434 30187931

[B17] Frazee A. C. Pertea G. Jaffe A. E. Langmead B. Salzberg S. L. Leek J. T. (2015). Ballgown bridges the gap between transcriptome assembly and expression analysis. *Nat. Biotechnol.* 33:243. 10.1038/nbt.3172 25748911 PMC4792117

[B18] Fu X.-Z. Zhang X.-Y. Qiu J.-Y. Zhou X. Yuan M. He Y.-Z. (2019). Whole-transcriptome RNA sequencing reveals the global molecular responses and ceRNA regulatory network of mRNAs, lncRNAs, miRNAs and circRNAs in response to copper toxicity in Ziyang Xiangcheng (*Citrus junos* Sieb. Ex Tanaka). *BMC Plant Biology* 19:509. 10.1186/s12870-019-2087-1 31752684 PMC6873749

[B19] Guo Y. Y. Tian S. S. Liu S. S. Wang W. Q. Sui N. (2018). Energy dissipation and antioxidant enzyme system protect photosystem II of sweet sorghum under drought stress. *Photosynthetica* 56 861–872. 10.1007/s11099-017-0741-0

[B20] He X. Guo S. Wang Y. Wang L. Shu S. Sun J. (2019). Systematic identification and analysis of heat−stress−responsive lncRNAs, circRNAs and miRNAs with associated co−expression and ceRNA networks in cucumber (*Cucumis sativus* L.). *Physiol. Plant.* 168 736–754. 10.1111/ppl.12997 31125116

[B21] Hsieh T.-H. Li C.-W. Su R.-C. Cheng C.-P. Tsai Y.-C. Chan M.-T. (2010). A tomato bZIP transcription factor, SlAREB, is involved in water deficit and salt stress response. *Planta* 231 1459–1473. 10.1007/s00425-010-1147-4 20358223

[B22] Hu H. Dai M. Yao J. Xiao B. Li X. Zhang Q. (2006). Overexpressing a NAM, ATAF, and CUC (NAC) transcription factor enhances drought resistance and salt tolerance in rice. *Proc. Natl. Acad. Sci. U.S.A.* 103 12987–12992. 10.1073/pnas.0604882103 16924117 PMC1559740

[B23] Huanca-Mamani W. Arias-Carrasco R. Cárdenas-Ninasivincha S. Rojas-Herrera M. Sepúlveda-Hermosilla G. Caris-Maldonado J. (2018). Long non-coding RNAs responsive to salt and boron stress in the hyper-arid Lluteno maize from Atacama desert. *Genes* 9:170. 10.3390/genes9030170 29558449 PMC5867891

[B24] Kang C.-H. Jung W. Kang Y. Kim J. Kim D. Jeong J. (2006). AtBAG6, a novel calmodulin-binding protein, induces programmed cell death in yeast and plants. *Cell Death Diff.* 13:84. 10.1038/sj.cdd.4401712 16003391

[B25] Kang J.-Y. Choi H.-I. Im M.-Y. Kim S. Y. (2002). *Arabidopsis* basic leucine zipper proteins that mediate stress-responsive abscisic acid signaling. *Plant Cell* 14 343–357. 10.1105/tpc.010362 11884679 PMC152917

[B26] Kim D. Pertea G. Trapnell C. Pimentel H. Kelley R. Salzberg S. L. (2013). TopHat2: accurate alignment of transcriptomes in the presence of insertions, deletions and gene fusions. *Genome Biol.* 14:R36. 10.1186/gb-2013-14-4-r36 23618408 PMC4053844

[B27] Kong L. Zhang Y. Ye Z.-Q. Liu X.-Q. Zhao S.-Q. Wei L. (2007). CPC: assess the protein-coding potential of transcripts using sequence features and support vector machine. *Nucleic Acids Res.* 35(suppl. 2) W345–W349. 10.1093/nar/gkm391 17631615 PMC1933232

[B28] Kumar V. Khare T. Shriram V. Wani S. H. (2018). Plant small RNAs: the essential epigenetic regulators of gene expression for salt-stress responses and tolerance. *Plant Cell Rep.* 37 61–75. 10.1007/s00299-017-2210-4 28951953

[B29] Langmead B. Salzberg S. L. (2012). Fast gapped-read alignment with Bowtie 2. *Nat. Methods* 9:357. 10.1038/nmeth.1923 22388286 PMC3322381

[B30] Leznicki P. High S. (2012). SGTA antagonizes BAG6-mediated protein triage. *Proc. Natl. Acad. Sci. U.S.A.* 109 19214–19219. 10.1073/pnas.1209997109 23129660 PMC3511132

[B31] Li Z. An X. Zhu T. Yan T. Wu S. Tian Y. (2019). Discovering and constructing ceRNA-miRNA-target gene regulatory networks during anther development in maize. *Int. J. Mol. Sci.* 20:3480. 10.3390/ijms20143480 31311189 PMC6678786

[B32] Liang W. Ma X. Wan P. Liu L. (2018). Plant salt-tolerance mechanism: a review. *Biochem. Biophys. Res. Commun.* 495 286–291. 10.1016/j.bbrc.2017.11.043 29128358

[B33] Maor R. (2017). MicroRNA compositions and methods for enhancing plant resistance to abiotic stress. Google Patents.

[B34] Matsui A. Seki M. (2019). “The involvement of long noncoding RNAs in response to plant stress,” in *Plant Long Non-Coding RNAs*, eds Chekanova A. J. Wang V. H.-L. (Berlin: Springer), 151–171. 10.1007/978-1-4939-9045-0_8 30945183

[B35] Meng X. Zhang P. Chen Q. Wang J. Chen M. (2018). Identification and characterization of ncRNA-associated ceRNA networks in *Arabidopsis* leaf development. *BMC Genomics* 19:607. 10.1186/s12864-018-4993-2 30103673 PMC6090674

[B36] Mercer T. R. Dinger M. E. Mattick J. S. (2009). Long non-coding RNAs: insights into functions. *Nat. Rev. Genet.* 10 155–159. 10.1038/nrg2521 19188922

[B37] Mondal T. K. Panda A. K. Rawal H. C. Sharma T. R. (2018). Discovery of microRNA-target modules of African rice (*Oryza glaberrima*) under salinity stress. *Sci. Rep.* 8:570. 10.1038/s41598-017-18206-z 29330361 PMC5766505

[B38] Moskovitz J. Berlett B. S. Poston J. M. Stadtman E. R. (1997). The yeast peptide-methionine sulfoxide reductase functions as an antioxidant in vivo. *Proc. Natl. Acad. Sci. U.S.A.* 94 9585–9589. 10.1073/pnas.94.18.9585 9275166 PMC23225

[B39] Oh J.-E. Hong S.-W. Lee Y. Koh E.-J. Kim K. Seo Y. W. (2005). Modulation of gene expressions and enzyme activities of methionine sulfoxide reductases by cold, ABA or high salt treatments in *Arabidopsis*. *Plant Sci.* 169 1030–1036. 10.1016/j.plantsci.2005.05.033

[B40] Pertea M. Pertea G. M. Antonescu C. M. Chang T.-C. Mendell J. T. Salzberg S. L. (2015). StringTie enables improved reconstruction of a transcriptome from RNA-seq reads. *Nat. Biotechnol.* 33:290. 10.1038/nbt.3122 25690850 PMC4643835

[B41] Powell B. Graham L. A. Stevens T. H. (2000). Molecular characterization of the yeast vacuolar H+-ATPase proton pore. *J. Biol. Chem.* 275 23654–23660. 10.1074/jbc.M004440200 10825180

[B42] Qin T. Xiong L. (2019). “Subcellular localization and functions of plant lncRNAs in drought and salt stress tolerance,” in *Plant Long Non-Coding RNAs*, eds Chekanova A. J. Wang V. H.-L. (Berlin: Springer), 173–186. 10.1007/978-1-4939-9045-0_9 30945184

[B43] Qu J. Li M. Zhong W. Hu C. (2015). Competing endogenous RNA in cancer: a new pattern of gene expression regulation. *Int. J. Clin. Exp. Med.* 8 17110–17116. 26770304 PMC4694204

[B44] Rizvi A. Z. Dhusia K. (2019). Computational mapping of the differentially expressed gene-lncRNA pairs present at the root nodule developmental stages of *Arachis hypogaea*. *bioRxiv*[Preprint]

[B45] Scharf K.-D. Berberich T. Ebersberger I. Nover L. (2012). The plant heat stress transcription factor (Hsf) family: structure, function and evolution. *Biochim. Biophys. Acta* 1819 104–119. 10.1016/j.bbagrm.2011.10.002 22033015

[B46] Seitz H. (2009). Redefining microRNA targets. *Curr. Biol.* 19 870–873. 10.1016/j.cub.2009.03.059 19375315

[B47] Shi H. Kim Y. Guo Y. Stevenson B. Zhu J.-K. (2003). The *Arabidopsis* SOS5 locus encodes a putative cell surface adhesion protein and is required for normal cell expansion. *Plant Cell* 15 19–32. 10.1105/tpc.007872 12509519 PMC143448

[B48] Song J. Wang B. (2015). Using euhalophytes to understand salt tolerance and to develop saline agriculture: Suaeda salsa as a promising model. *Ann. Bot.* 115 541–553. 10.1093/aob/mcu194 25288631 PMC4332605

[B49] Sui N. Han G. (2014). Salt-induced photoinhibition of PSII is alleviated in halophyte *Thellungiella halophila* by increases of unsaturated fatty acids in membrane lipids. *Acta Physiol. Plant.* 36 983–992. 10.1007/s11738-013-1477-5

[B50] Sui N. Yang Z. Liu M. Wang B. (2015). Identification and transcriptomic profiling of genes involved in increasing sugar content during salt stress in sweet sorghum leaves. *BMC Genomics* 16:534. 10.1186/s12864-015-1760-5 26186930 PMC4506618

[B51] Swiezewski S. Liu F. Magusin A. Dean C. (2009). Cold-induced silencing by long antisense transcripts of an *Arabidopsis* Polycomb target. *Nature* 462:799. 10.1038/nature08618 20010688

[B52] Tariq M. Paszkowski J. (2004). DNA and histone methylation in plants. *Trends Genet.* 20 244–251. 10.1016/j.tig.2004.04.005 15145577

[B53] Ueda M. Sako K. Seki M. (2018). “Regulation and modification of the epigenome for enhanced salinity tolerance in crop plants,” in *Salinity Responses and Tolerance in Plants*, Vol. 2 eds Kumar V. Wani S. H. Suprasanna P. Tra006E L.-S. P. (Berlin: Springer), 77–91. 10.1007/978-3-319-90318-7_4

[B54] Uno Y. Furihata T. Abe H. Yoshida R. Shinozaki K. Yamaguchi-Shinozaki K. (2000). *Arabidopsis* basic leucine zipper transcription factors involved in an abscisic acid-dependent signal transduction pathway under drought and high-salinity conditions. *Proc. Natl. Acad. Sci. U.S.A.* 97 11632–11637. 10.1073/pnas.190309197 11005831 PMC17252

[B55] Vera-Estrella R. Barkla B. J. García-Ramírez L. Pantoja O. (2005). Salt stress in *Thellungiella halophila* activates Na+ transport mechanisms required for salinity tolerance. *Plant Physiol.* 139 1507–1517. 10.1104/pp.105.067850 16244148 PMC1283785

[B56] Vissenberg K. Fry S. C. Verbelen J.-P. (2001). Root hair initiation is coupled to a highly localized increase of xyloglucan endotransglycosylase action in *Arabidopsis* roots. *Plant Physiol.* 127 1125–1135. 10.1104/pp.127.3.1125 11706192 PMC129281

[B57] Wang T.-Z. Liu M. Zhao M.-G. Chen R. Zhang W.-H. (2015). Identification and characterization of long non-coding RNAs involved in osmotic and salt stress in *Medicago truncatula* using genome-wide high-throughput sequencing. *BMC Plant Biol.* 15:131. 10.1186/s12870-015-0530-5 26048392 PMC4457090

[B58] Wang Z. Li B. Li Y. Zhai X. Dong Y. Deng M. (2018). Identification and characterization of long noncoding RNA in *Paulownia tomentosa* treated with methyl methane sulfonate. *Physiol. Mol. Biol. Plants* 24 325–334. 10.1007/s12298-018-0513-8 29515326 PMC5834995

[B59] Wassenegger M. Heimes S. Riedel L. Sänger H. L. (1994). RNA-directed de novo methylation of genomic sequences in plants. *Cell* 76 567–576. 10.1016/0092-8674(94)90119-8 8313476

[B60] Wu H.-J. Wang Z.-M. Wang M. Wang X.-J. (2013). Widespread long noncoding RNAs as endogenous target mimics for microRNAs in plants. *Plant Physiol.* 161 1875–1884. 10.1104/pp.113.215962 23429259 PMC3613462

[B61] Wu Y. Thorne E. T. Sharp R. E. Cosgrove D. J. (2001). Modification of expansin transcript levels in the maize primary root at low water potentials. *Plant Physiol.* 126 1471–1479. 10.1104/pp.126.4.1471 11500546 PMC117147

[B62] Xin M. Wang Y. Yao Y. Song N. Hu Z. Qin D. (2011). Identification and characterization of wheat long non-protein coding RNAs responsive to powdery mildew infection and heat stress by using microarray analysis and SBS sequencing. *BMC Plant Biol.* 11:61. 10.1186/1471-2229-11-61 21473757 PMC3079642

[B63] Yang Z. Wang Y. Wei X. Zhao X. Wang B. Sui N. (2017). Transcription profiles of genes related to hormonal regulations under salt stress in sweet sorghum. *Plant Mol. Biol. Rep.* 35 586–599. 10.1007/s11105-017-1047-x

[B64] Yang Z. Yang C. Wang Z. Yang Z. Chen D. Wu Y. (2019). LncRNA expression profile and ceRNA analysis in tomato during flowering. *PloS One* 14:e0210650. 10.1371/journal.pone.0210650 30653557 PMC6336255

[B65] Yang Z. Zheng H. Wei X. Song J. Wang B. Sui N. (2018). Transcriptome analysis of sweet *Sorghum* inbred lines differing in salt tolerance provides novel insights into salt exclusion by roots. *Plant Soil* 430 423–439. 10.1007/s11104-018-3736-0

[B66] Yuan F. Bingying L. Baoshan W. (2016). Progress in studying salt secretion from the salt glands in recretohalophytes: how do plants secrete salt? *Front. Plant Sci.* 7:977. 10.3389/fpls.2016.00977 27446195 PMC4927796

[B67] Yvonne T. John R. Pier Paolo P. (2014). The multilayered complexity of ceRNA crosstalk and competition. *Nature* 505 344–352. 10.1038/nature12986 24429633 PMC4113481

[B68] Zhang J. Wei L. Jiang J. Mason A. S. Li H. Cui C. (2018). Genome-wide identification, putative functionality and interactions between lncRNAs and miRNAs in *Brassica* species. *Sci. Rep.* 8:4960. 10.1038/s41598-018-23334-1 29563515 PMC5862966

[B69] Zhang L. Zheng Y. Jagadeeswaran G. Li Y. Gowdu K. Sunkar R. (2011). Identification and temporal expression analysis of conserved and novel microRNAs in *Sorghum*. *Genomics* 98 460–468. 10.1016/j.ygeno.2011.08.005 21907786

[B70] Zhang Q. Zhao C. Li M. Sun W. Zhao Y. (2013). Genome-wide identification of *Thellungiella salsuginea* microRNAs with putative roles in the salt stress response. *BMC Plant Biol.* 13:180. 10.1186/1471-2229-13-180 24237587 PMC4225614

[B71] Zhang Y. Li X. Goodrich J. Wu C. Wei H. Yang S. (2016). Reduced function of the RNA-binding protein FPA rescues a T-DNA insertion mutant in the *Arabidopsis* ZHOUPI gene by promoting transcriptional read-through. *Plant Mol. Biol.* 91 549–561. 10.1007/s11103-016-0487-2 27164978

[B72] Zhu J. Dong C.-H. Zhu J.-K. (2007). Interplay between cold-responsive gene regulation, metabolism and RNA processing during plant cold acclimation. *Curr. Opin. plant Biol.* 10 290–295. 10.1016/j.pbi.2007.04.010 17468037

