# Supplementary figures and images for: Comparative Transcriptome Analysis Reveals New lncRNAs Responding to Salt Stress in Sweet Sorghum
Source: Front Bioeng Biotechnol. 2020 Apr 15;8:331. doi: 10.3389/fbioe.2020.00331 (PMC7174691; doi:10.3389/fbioe.2020.00331)

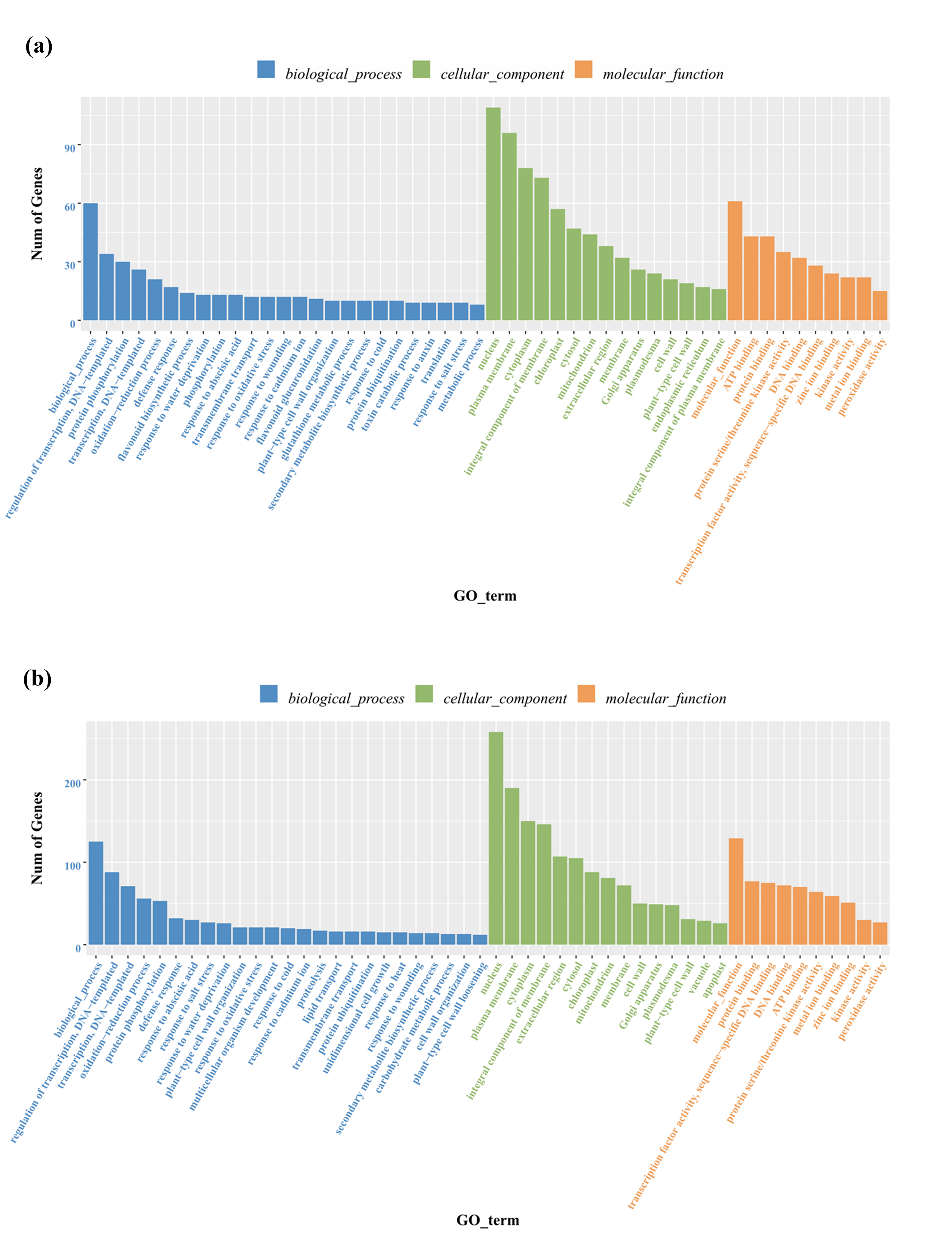

Supplement: FIGURE S1 — Functional enrichment analysis of differentially expressed genes in M-81E (A) and Roma (B). [file Image_1.TIF]

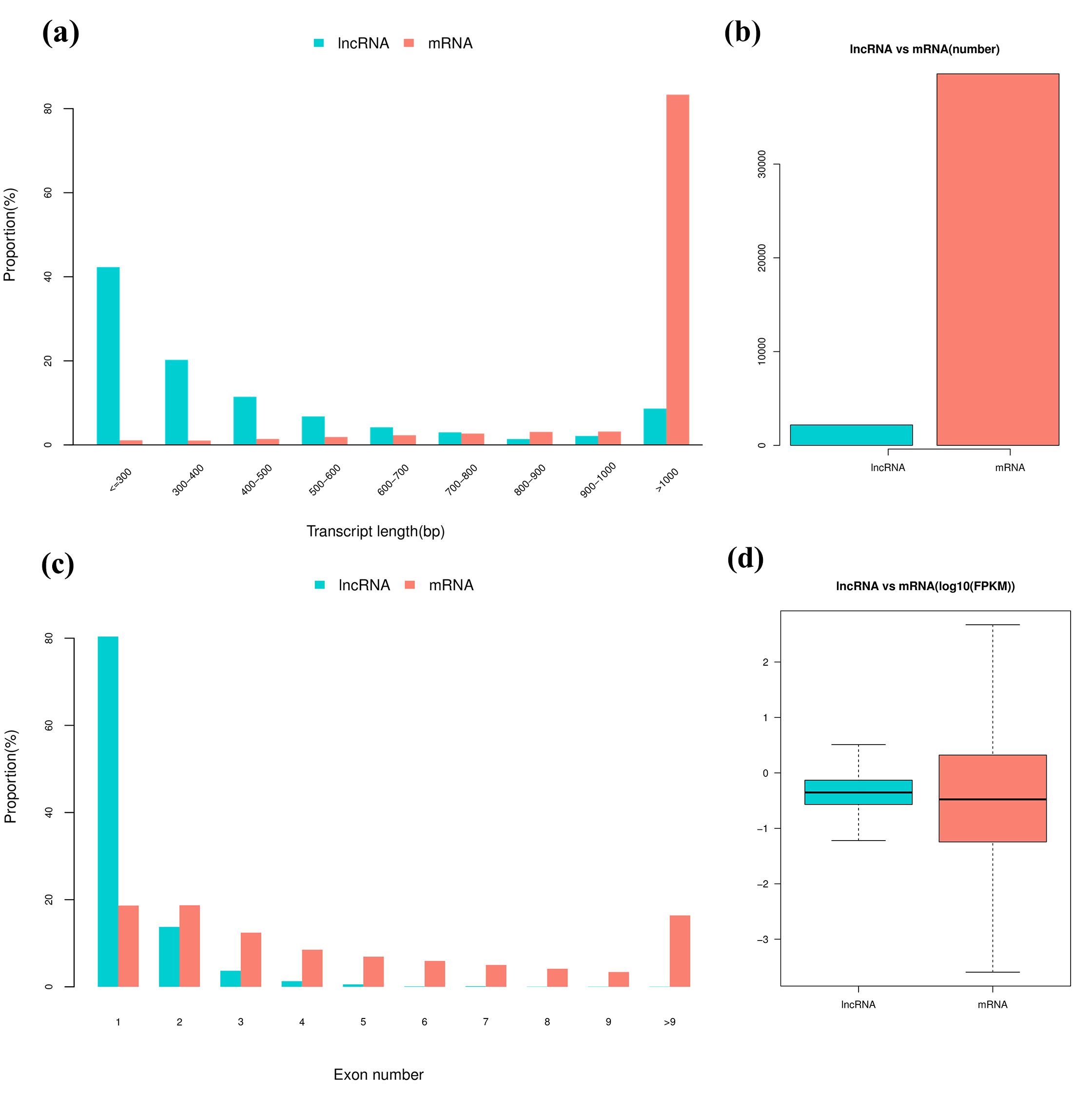

Supplement: FIGURE S2 — Comparison of lncRNA and mRNA of the transcript length, number, exon number and expression levels. (A) The length of lncRNA and mRNA. The x-axis represents the length of the transcript, and the y-axis represents the percentage of lncRNA and mRNA, respectively. (B) The number of lncRNA and mRNA. (C) The exon number of lncRNA and mRNA. (D) The expression levels of lncRNA and mRNA. [file Image_2.TIF]

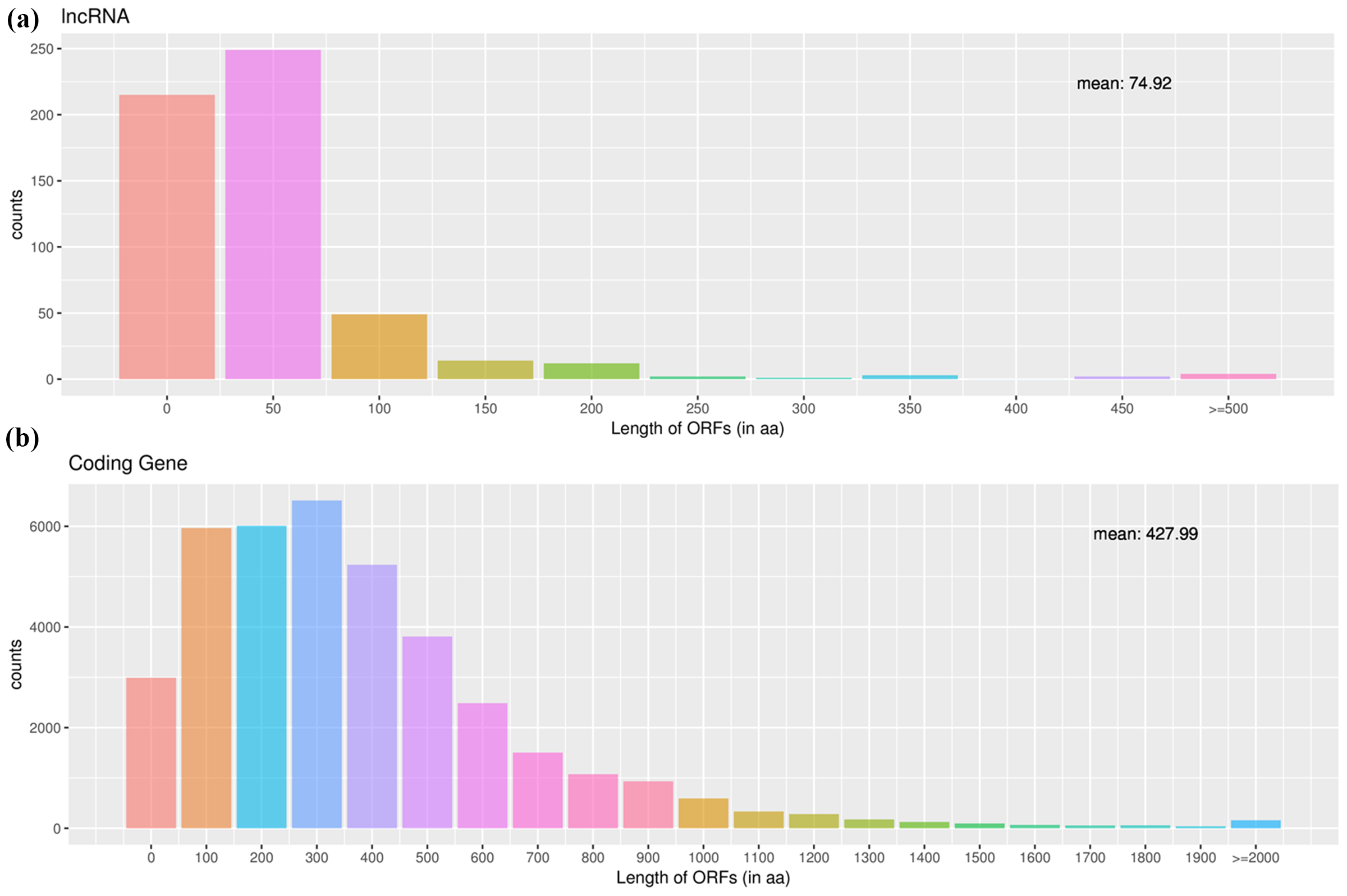

Supplement: FIGURE S3 — Comparison of lncRNA (A) and mRNA (B) of open reading frames. [file Image_3.TIF]

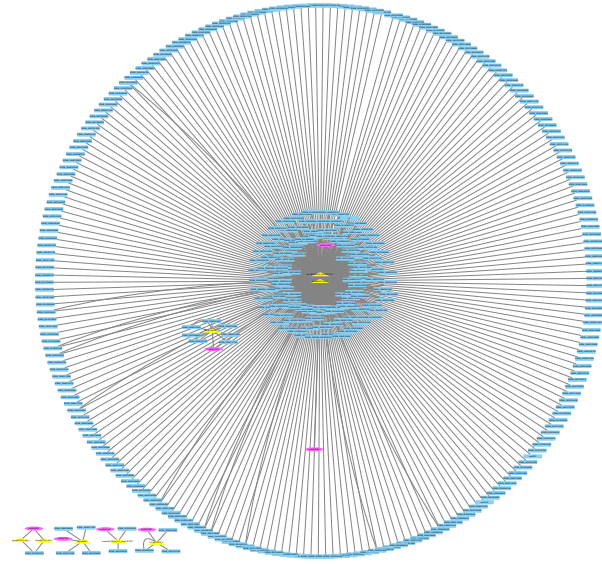

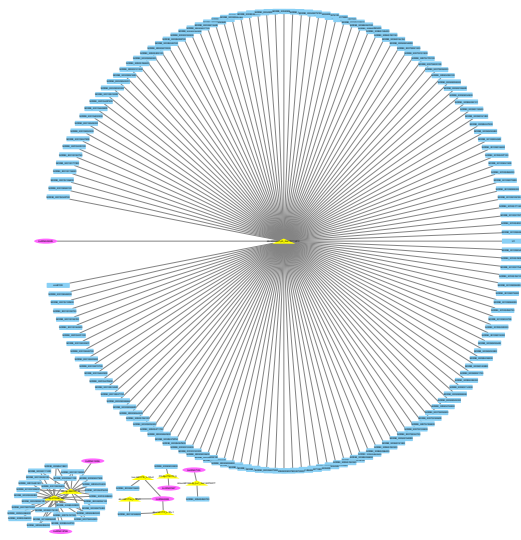

Supplement: FIGURE S4 — The global ceRNA network in the two sweet sorghum strains. Red, yellow, and blue nodes represent lncRNAs, miRNAs, and mRNAs, respectively. [file Image_4.pdf]
