# Supplementary material for: Comparative Transcriptome Analysis Reveals New lncRNAs Responding to Salt Stress in Sweet Sorghum
Source: Front Bioeng Biotechnol. 2020 Apr 15;8:331. doi: 10.3389/fbioe.2020.00331 (PMC7174691; doi:10.3389/fbioe.2020.00331)
Supplement: TABLE S1 — Primer sequences for qRT-PCR of 5 lncRNAs, 5 miRNAs and 14 mRNAs. [file Table_1.docx]

**Supplemental Table 1** Primer sequences for qRT-PCR of 5 lncRNAs, 5 miRNAs and 14 mRNAs.

| **RNA** | **Primer sequences** |
| --- | --- |
| lncRNA11310-F | TATGCATCCACAATGCGCGG |
| lncRNA11310-R | CGCAAACAGACAAAAGCGGA |
| lncRNA13472-F | GTACACATGGTGGCGGTTGA |
| lncRNA13472-R | ACTCACGAAGCAAGCGATGT |
| lncRNA2846-F | ACAAGTTAGTGCCGCTTGGT |
| lncRNA2846-R | CCGTGTGAGCAAATGGGGAT |
| lncRNA26929-F | GCAAGATTCGATGTGACGGGA |
| lncRNA26929-R | ATTTGAGGTAGCTGTGGACTACT |
| lncRNA14798-F | TACGTCTCCGATCCAGTTCC |
| lncRNA14798-R | AGTGCGGAGCGGATAATACA |
| sbi-MIR5567-p3-2ss16CT17TC-F | GCAAATTTTTCAGATTCTCC |
| sbi-MIR5567-p3-2ss16CT17TC-R | TGCGTGTCGTGGAGTC |
| sbi-MIR169b-p3-F | GTCTCCGGCAAGTTGT |
| sbi-MIR169b-p3-R | TGCGTGTCGTGGAGTC |
| sbi-MIR5567-p5-2-F | GCATGCATGGAGTATTAAA |
| sbi-MIR5567-p5-2-R | TGCGTGTCGTGGAGTC |
| sbi-MIR5567-p5-2ss17CT18TC-F | GCGAAAATTTTTCAGATTC |
| sbi-MIR5567-p5-2ss17CT18TC-R | TGCGTGTCGTGGAGTC |
| PC-3p-270284-34-F | AGTTCCACTCCAATCCAC |
| PC-3p-270284-34-R | TGCGTGTCGTGGAGTC |
| SORBI_3010G218400-F | GTTTACGTGGGTTGTGGGGA |
| SORBI_3010G218400-R | TCACGTCCATCAGGCAATGT |
| SORBI_3009G182800-F | CCAGCAGTTGCGGATGTTTC |
| SORBI_3009G182800-R | CAGCGAGAGGATCAGAACTCA |
| SORBI_3003G327000-F | AGGCTGAGGTGGTGAGGTTA |
| SORBI_3003G327000-R | GCTCCGATGCTTTTGGAACG |
| SORBI_3001G158100-F | ATTCGTGGACTTTGCGGTGA |
| SORBI_3001G158100-R | TCACATCGAACCTGACGGTG |
| SORBI_3001G223100-F | CGTGTACAACGCCGAGGTAT |
| SORBI_3001G223100-R | TATAGGGCCACCTCCACTGC |
| SORBI_3010G081800-F | GCTGAGGTGGCAAAACTCAAG |
| SORBI_3010G081800-R | TGCCTTTGGTCCATGTTGGT |
| SORBI_3007G046900-F | CTTGTGCGGGGTCATAGTCA |
| SORBI_3007G046900-R | GAATCCAGTAGGTGGCCCAG |
| SORBI_3006G123500-F | GTACCGGACGGGCATCTACT |
| SORBI_3006G123500-R | GACGATCTTGTCCTCCCACT |
| SORBI_3009G042700-F | CCAACTCGCTCTGTCCATCG |
| SORBI_3009G042700-R | GGCACAAAGGGAACGAAACC |
| SORBI_3002G302000-F | GACCTGCGGTAGTGGTTTCA |
| SORBI_3002G302000-R | ACAGGTAGAAGGCGGTGTTG |
| SORBI_3002G237000-F | GTGCGTTCCTGCCAAAATACAA |
| SORBI_3002G237000-R | GGCCCGTTGTTGGACTTGAG |
| SORBI_3004G116300-F | AGAACATCCCACTGACAACCA |
| SORBI_3004G116300-R | TGCTGGTTTCTCTTCTTCAGTCA |
| SORBI_3004G302400-F | CGTCTCAGCTCTTCAGCCAC |
| SORBI_3004G302400-R | GAGGCGTAGTCCAGTGCTTC |
| SORBI_3009G208000-F | TGTCTCCGATCCGCCTTTTG |
| SORBI_3009G208000-R | GGGTTGCCCTCTGGAAGAAA |
| U6-F | CGATAAAATTGGAACGATACAGA |
| U6-R | ATTTGGACCATTTCTCGATTTGT |
| sbi-actin-F | ACGGCCTGGATGGCGACGTACATG |
| sbi-actin-R | GCAGAAGGACGCCTACGTTGGTGAC |

**Supplemental Table 2** Statistical data of the RNA-seq reads for two samples.

| **Samples** | **Raw Data** | | **Valid Data** | | **Valid Ratio** | **Q20%** | **Q30%** | **GC content%** |
| --- | --- | --- | --- | --- | --- | --- | --- | --- |
|  | Read | Base | Read | Base |  |  |  |  |
| M-81E-CK | 90487489 | 13.57G | 89544103 | 13.43G | 98.96 | 99.85 | 98.71 | 52.67 |
| M-81E-salt | 89357725 | 13.4G | 88662485 | 13.3G | 99.21 | 99.71 | 96.77 | 52.5 |
| Roma-CK | 90841700 | 13.63G | 89279592 | 13.39G | 98.28 | 99.68 | 98.23 | 52.5 |
| Roma-salt | 92540189 | 13.88G | 90733094 | 13.61G | 98.05 | 99.55 | 98.06 | 53.17 |

**Supplemental Table 3** LncRNA-miRNA-mRNA relationship pairs and functional annotation of DEGs.

| **Strain** | **lncRNA** | **miRNA** | **Gene ID** | **chr** | **strand** | **start** | **end** | **Gene name** | **Log_2_(fc)** | **P value** | **Regulation** |
| --- | --- | --- | --- | --- | --- | --- | --- | --- | --- | --- | --- |
| **M-81E** | lncRNA13472 | sbi-MIR169b-p3 | SORBI-3010G218400 | chr10 | - | 56112585 | 56120718 | VHA-A | 1.869228 | 0.38316 | Up |
|  | lncRNA11310 | sbi-MIR5567-p3-2ss16CT17TC | SORBI-3001G158100 | chr1 | + | 12820997 | 12824714 | BPM1 | 1.249456 | 0.2817 | Up |
|  |  |  | SORBI-3001G223100 | chr1 | - | 21334060 | 21340341 | NPF5.2 | 1.043764 | 0.001697 | Up |
|  |  |  | SORBI-3002G237000 | chr2 | \| + \| \| --- \| | 62743579 | 62745574 | FLA1 | -1.964330 | 0.048565 | Down |
|  |  |  | SORBI-3002G302000 | chr2 | + | 67844329 | 67848475 | XTH31 | 1.222253 | 0.000263 | Up |
|  |  |  | SORBI-3003G327000 | chr3 | + | 65278175 | 65282100 | bZIP23 | inf | 0.050197 | Up |
|  |  |  | SORBI-3009G182800 | chr9 | - | 53632420 | 53636146 | bZIP23 | inf | 0.4084 | Up |
|  | lncRNA2846 | sbi-MIR5567-p5-2 | SORBI-3001G158100 | chr1 | + | 12820997 | 12824714 | BPM1 | 1.249456 | 0.2817 | Up |
|  |  |  | SORBI-3001G223100 | chr1 | - | 21334060 | 21340341 | NPF5.2 | 1.043764 | 0.001697 | Up |
|  |  |  | SORBI-3002G237000 | chr2 | + | 62743579 | 62745574 | FLA1 | -1.964330 | 0.048565 | Down |
|  |  |  | SORBI-3002G302000 | chr2 | + | 67844329 | 67848475 | XTH31 | 1.222253 | 0.000263 | Up |
|  |  |  | SORBI-3003G327000 | chr3 | + | 65278175 | 65282100 | bZIP23 | inf | 0.050197 | Up |
|  |  |  | SORBI-3004G116300 | chr4 | + | 12145243 | 12167088 | BAG6 | 2.324406 | 0.023039 | Up |
|  |  |  | SORBI-3004G302400 | chr4 | + | 64103336 | 64112306 | NAC007 | 1.488383 | 0.006880 | Up |
|  |  |  | SORBI-3006G123500 | chr6 | - | 48915680 | 48917326 | MSRA2-1 | 1.976440 | 0.26058 | Up |
|  |  |  | SORBI-3007G046900 | chr7 | - | 4684838 | 4688008 | BPM1 | 1.718159 | 0.49244 | Up |
|  |  |  | SORBI-3009G182800 | chr9 | - | 53632420 | 53636146 | bZIP23 | inf | 0.4084 | Up |
|  |  |  | SORBI-3009G208000 | chr9 | + | 55539190 | 55543184 | HSFA4D | -1.137883 | 0.038020 | Down |
|  |  |  | SORBI-3010G081800 | chr10 | + | 6998322 | 7004279 | bZIPTF23 | 2.427835 | 0.103978 | Up |
| **Roma** | lncRNA26929 | sbi-MIR5567-p5-2ss17CT18TC | SORBI-3003G327000 | chr3 | + | 65278175 | 65282100 | bZIP23 | 2.283570 | 0.42264 | Up |
|  |  |  | SORBI-3001G158100 | chr1 | + | 12820997 | 12824714 | BPM1 | 2.607521 | 0.2302 | Up |
|  |  |  | SORBI-3001G223100 | chr1 | - | 21334060 | 21340341 | NPF5.2 | -1.440500 | 0.29476 | Down |
|  | lncRNA14798 | PC-3p-270284-34 | SORBI-3009G042700 | chr9 | + | 4071948 | 4078347 | NHX2 | -1.713690 | 0.120721 | Down |

**Supplemental Table 4** Predicted lncRNAs in ceRNA networks.

| **lncRNA** | **class_code** | **chr** | **strand** | **start** | **end** | **length** | **log_2_(fc)** | **Pvalue** | **Regulation** |
| --- | --- | --- | --- | --- | --- | --- | --- | --- | --- |
| lncRNA13472 | u | chr3 | . | 67264519 | 67267567 | 3049 | 1.16 | 0.00 | up |
| lncRNA11310 | u | chr3 | . | 10752043 | 10753294 | 1252 | 1.92 | 0.00 | up |
| lncRNA2846 | u | chr1 | - | 63170391 | 63172014 | 1624 | 1.60 | 0.23 | up |
| lncRNA26929 | u | chr9 | . | 57221184 | 57223432 | 2249 | -2.01 | 0.14 | down |
| lncRNA14798 | i | chr4 | . | 5613624 | 5614091 | 468 | -3.19 | 0.13 | down |

**Supplemental Table 5** Predicted miRNAs in ceRNA networks.

| **miRNA ID** | **miR-seq** | **chr** | **start** | **end** | **length** | **Sequence in miRbase** | **Regulation** |
| --- | --- | --- | --- | --- | --- | --- | --- |
| sbi-MIR169b-p3 | TCTCCGGCAAGTTGTCCTTGG | chr10 | 56120427 | 56120528 | 21 | New | up |
| sbi-MIR5567-p3-2ss16CT17TC | AAATTTTTCAGATTCTCCGTCACA | chr2 | 15423539 | 15423589 | 24 | New | up |
| sbi-MIR5567-p5-2 | ATGCATGGAGTATTAAATATAGA | chr1 | 64615524 | 64615576 | 23 | New | up |
| sbi-MIR5567-p5-2ss17CT18TC | AAAATTTTTCAGATTCTCCGTC | chr4 | 45400269 | 45400403 | 22 | New | up |
| PC-3p-270284-34 | AGTTCCACTCCAATCCACACCAAC | chr6 | 50156858 | 50157004 | 24 | New | up |
|  |  | chr9 | 5575925 | 5576085 |  |  |  |
